# Hearing problems in patients with hereditary gelsolin amyloidosis

**DOI:** 10.1186/s13023-021-02077-9

**Published:** 2021-10-24

**Authors:** Tuuli Mustonen, Ville Sivonen, Sari Atula, Sari Kiuru-Enari, Saku T. Sinkkonen

**Affiliations:** 1 Clinical Neurosciences, Neurology, Helsinki University Hospital and University of Helsinki, HYKS, Tornisairaala, Neupkl, Haartmaninkatu 4, 00029 Helsinki, Finland; 2 Department of Otorhinolaryngology – Head and Neck Surgery, Head and Neck Center, Helsinki University Hospital and University of Helsinki, Helsinki, Finland

**Keywords:** Gelsolin amyloidosis, Automated audiometry, Speech-in-noise, Speech Spatial and Qualities of Hearing Scale, Hearing loss

## Abstract

**Background:**

Gelsolin amyloidosis (AGel amyloidosis) is a hereditary form of systemic amyloidosis featuring ophthalmological, neurological and cutaneous symptoms. Previous studies based mainly on patients’ self-reporting have indicated that hearing impairment might also be related to the disease, considering the progressive cranial neuropathy characteristic for AGel amyloidosis. In order to deepen the knowledge of possible AGel amyloidosis-related hearing problems, a clinical study consisting of the Speech, Spatial and Qualities of Hearing Scale (SSQ) questionnaire, clinical examination, automated pure-tone audiometry and a speech-in-noise test was designed.

**Results:**

Of the total 46 patients included in the study, eighteen (39%) had self-reported hearing loss. The mean scores in the SSQ were 8.2, 8.3 and 8.6 for the Speech, Spatial and Qualities subscales, respectively. In audiometry, the mean pure tone average (PTA) was 17.1 (SD 12.2) and 17.1 (SD 12.3) dB HL for the right and left ears, respectively, with no difference to gender- and age-matched, otologically normal reference values. The average speech reception threshold in noise (SRT) was − 8.2 (SD 1.5) and − 8.0 (SD 1.7) dB SNR for the right and left ears, respectively, which did not differ from a control group with a comparable range in PTA thresholds.

**Conclusion:**

Although a significant proportion of AGel amyloidosis patients experience subjective difficulties in hearing there seems to be no peripheral or central hearing impairment at least in patients up to the age of 60 years.

## Introduction

Gelsolin amyloidosis (AGel amyloidosis) is a rare systemic amyloid disease with a fully penetrant autosomal dominant inheritance pattern [1]. The disease is mainly found in Finland, yet individual cases or kindreds have been reported worldwide [1–6]. AGel amyloidosis is caused by different point mutations in the gelsolin gene, c.640G>A being the major one and so far, the only mutation reported in Finland [1, 7, 8]. Misfolding and abnormal proteolytic cleavage of mutant plasma gelsolin lead eventually to the formation of amyloid oligomers and further, deposition of mature gelsolin amyloid (AGel) fibrils, which both probably contribute to the disease phenotype [1, 9]. Recently, an alternative non-proteolytic oligomerisation mechanism has also been suggested [10].

The clinical picture of AGel amyloidosis is dominated by ophthalmological, neurological, and cutaneous symptoms causing considerable disease burden [11–13]. Gelsolin type of corneal lattice amyloidosis is usually the first manifestation of the disease [14]. Recurrent corneal erosions may cause visual impairment and lead even to blindness [1] in addition to variety of other ophthalmological symptoms [11]. Slowly progressive cranial neuropathy is the most significant neurological sign. Uni- or bilateral facial palsy is considered as a hallmark of AGel amyloidosis but involvement of other cranial nerves, including vestibulocochlear nerve, has been observed as well [1, 12]. Both the facial nerve paresis and cutis laxa (abnormal laxity of skin) compromise oral functions and contribute to a lack of expression giving a mask-like impression [13]. Mostly sensory polyneuropathy and mild autonomic nervous system involvement are common [14, 15]. Clinically significant cardiac or renal involvement among heterozygotes is rare but possible [16–19]. The disease is age-related as the patients become symptomatic on average around the age of 40 years [20]. However, pre-symptomatic findings, mostly ocular, have been demonstrated earlier, already under the age of 30 years [14, 20, 21]. The disease does not shorten the lifespan [22, 23].

Otological involvement of amyloidosis is extremely rare [24]. Yet recently, in case of wild type (ATTRwt) and hereditary transthyrethin (ATTRv) amyloidosis, sensorineural hearing loss was discovered to be associated with both diseases [25, 26]. In AGel amyloidosis, 39% of the patients in the Finnish Gelsolin Amyloidosis Patient Registry (FIN-GAR) [20] reported impaired hearing. Earlier studies and case reports have suggested that AGel amyloidosis might also cause hearing loss [12, 14, 21, 27–30] based however only on self-reporting, tuning fork tests and individual audiograms. The methods used in this study, the Speech, Spatial and Qualities of Hearing Scale (SSQ) [31], pure tone audiometry and a speech-in-noise test [32] are related but also complementary as they measure hearing from different perspectives. A greater pure tone average (PTA) in pure tone audiometry indicating hearing impairment has been reported to result in poorer SSQ scores [31] as well as a poorer performance in a speech-in-noise test [33]. This study aims to collect comprehensive audiological information and to investigate the possible hearing impairment in a cohort of AGel amyloidosis patients.

## Results

### Clinical findings

Altogether 46 patients (76% female, 24% male), of which 43 had been registered in FIN-GAR, were enrolled in the study. Additionally, three patients, aged 66 years, were recruited at a patient organization meeting. The mean age was 60 years (range 47–66 and median 61 years) at the time of examination. The AGel amyloidosis diagnosis was based on genetic testing in 32 (70%) of the patients. All the patients had the diagnosis confirmed by an ophthalmologist (demonstration of pathognomonic lattice corneal dystrophy type 2) and a positive family history. A total of 18 (39%) patients reported to have hearing impairment and 19 (41%) reported to have problems in speech discrimination in the presence of background noise. In total, 14 (30%) patients experienced both hearing loss and difficulties in speech discrimination. A hearing aid was used by four (9%) patients. All patients were symptomatic due to AGel amyloidosis (Table 1).

**Table 1:** Common gelsolin amyloidosis-related symptoms and signs reported by the patients (n = 46)

Symptom	n (%)
Eye dryness	45 (98%)
Corneal lattice dystrophy	26 (57%)
Impaired vision	39 (85%)
Facial palsy	42 (91%)
Numbness of hands or feet	32 (70%)
Myokymias	24 (52%)
Carpal tunnel syndrome	17 (37%)
Cutis laxa	31 67%)
Surgical face operation (one or many)	17 (37%)
Surgical eye operation (one or many)	22 (48%)
Carpal tunnel syndrome operation (one or many)	18 (39%)

In pneumatic otoscopy, all the patients had normal tympanic membrane with good mobility excluding one with air leakage due to poorly fitting aural speculum. The Rinne test was normal in all patients. In the Weber test, 10 (22%) patients reported lateralization. Clinical examination of the cranial nerves revealed facial nerve paresis or total paralysis (uni- or bilateral, one or many branches) in 43 (93%) patients. Clearly (symmetrical and disease-like) drooping eyelids were found in 26 (57%) patients, making it difficult to evaluate the function of the levator palpeprae superioris muscle. A total of five (11%) patients had decreased sensation on the facial area and one (2%) patient had no sensation in the cornea tested by a light touch by a wisp of cotton. Provocation of gag reflex was unsuccessful with four (9%) patients and turning of the head was weak on both sides with one (2%) patient. Tongue was furrowed and deviated in one (2%) patient and nine (20%) patients had an abnormally large and/or furrowed tongue. Objective dysarthria was found in three (7%) patients.

### The self-reported hearing disability

The SSQ questionnaire [31] was used to self-describe hearing-related difficulties. In the SSQ, the respondents reported their auditory performance from 0 (maximal disability) to 10 (no disability). The mean results for the different subscales were as follows: Speech 8.2 (SD 1.3, n = 44), Spatial 8.3 (SD 1.1, n = 44) and Qualities 8.6 (SD 1.0, n = 43) (Table 2) indicating on average normal hearing abilities [34]. Nine patients had an average response under 7 in some or all of SSQ subscales (Table 3).

**Table 2:** The mean values for the Speech, Spatial and Qualities of Hearing Scale questionnaire sub scales and objective hearing measurements among patients that report (YES) and do not report (NO) hearing loss

	All patients (n = 46**)	YES (n = 18**)	NO (n = 28**)	F	p value
Speech mean (SD)	8.2 (1.3)	7.1 (1.3)	8.8 (0.7)	30.277	< 0.0005***
Spatial mean (SD)	8.3 (1.1)	7.7 (1.1)	8.6 (1.0)	6.325	0.016
Qualities mean (SD)	8.6 (1.0)	8 (1.2)	9 (0.7)	12.213	0.001**
PTA mean, right (SD) dB	17.1 (12.2)	23.4 (11.9)	13 (10.7)	5.559	0.023
PTA mean, left (SD) dB	17.1 (12.3)	23.6 (12.8)	12.9 (10.1)	5.899	0.020
SRT mean, right (SD) dB SNR	− 8.2 (1.5)	− 7.8 (2.0)	− 8.5 (0.9)	0.435	0.513
SRT mean, left (SD) dB SNR	− 8.0 (1.7)	− 7.7 (1.9)	− 8.2 (1.5)	0.005	0.941

A univariate analysis of variance was conducted to compare the effect of reported hearing loss on each of the dependent variables described in the table

PTA = pure tone average (0, 5, 1, 2, 4 kHz), SRT = speech reception threshold in noise, SNR = signal-to-noise ratio

^**^The data for SSQ were missing for 2 patients in the subgroup “YES” and the qualities data were missing for one in the subgroup “NO”

^***^p-values less than 0.0071 are considered statistically significant after the Bonferroni correction

**Table 3:** The Speech, Spatial and Qualities of Hearing Scale questionnaire scores and objective hearing measurements for the nine patients that scored less than 7 in one or many of the subscales

Patient	Hearing device	SSQ			PTA (dB)		SRT (dB SNR)	
		Speech	Spatial	Qualities	Right	Left	Right	Left
1	NO	4.9	5.0	5.6	15	16.25	− 9.3	− 9.4
2	YES	4.3	7.3	5.8	45	51.25	− 4.2	− 5.0
3	NO	8.4	5.6	8.6	17.5	22.5	− 8.7	− 6.9
4	YES	5.8	7.2	7.9	28.75	32.5	− 7.7	− 6.3
5	NO	6.8	6.8	7.5	17.5	21.25	− 9.0	− 8.8
6	NO	7.5	7.5	6.7	5	6.25	− 8.3	− 8.3
7	NO	7.6	6.7	8.5	10	3.75	− 8.9	− 8.8
8	NO	6.0	7.8	8.3	21.25	26.25	− 8.9	− 6.8
9	NO	7.8	6.5	9.4	8.75	10	− 8.0	− 9.0

SSQ = The Speech, Spatial and Qualities of Hearing Scale, PTA = pure tone average (0, 5, 1, 2, 4 kHz), SRT = speech reception threshold in noise, SNR = signal-to-noise ratio

### Pure-tone audiometry

**Fig. 1:**
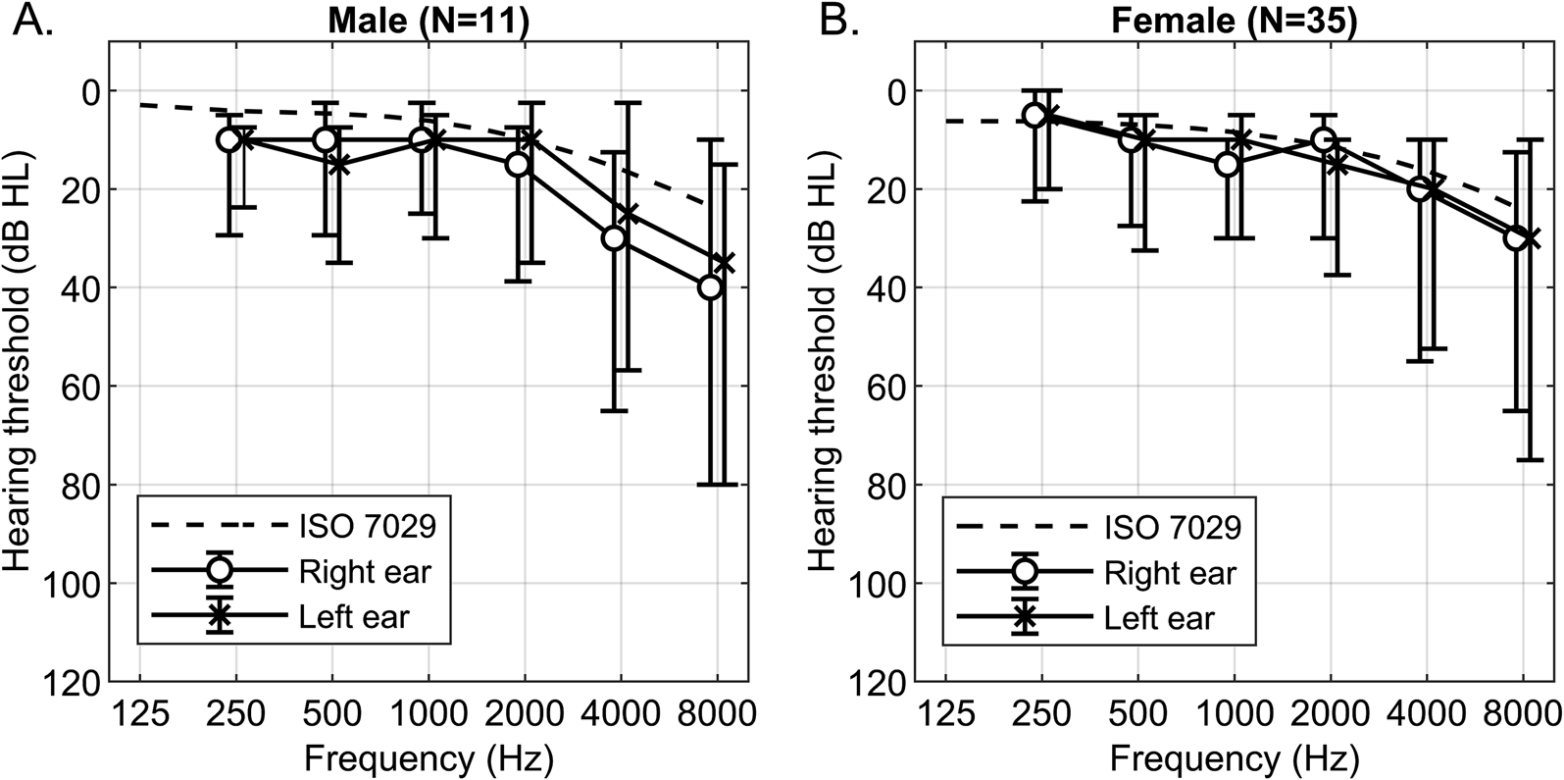
The median air-conduction thresholds and interquartile ranges for male (**A**) and female (**B**) subgroups compared with the age-matched ISO 7029 standard

Automated pure-tone audiometry was performed to assess hearing sensitivity and to identify conductive and/or sensorineural hearing loss. Figure 1 depicts the median hearing thresholds of AGel amyloidosis patients compared with the median hearing thresholds based on gender- and age-matched otologically normal reference values [35]. The mean pure tone average (PTA) for the patients was 17.1 (SD 12.2) and 17.1 (SD 12.3) dB HL for the right and left ears, respectively. There were no statistically significant differences in PTAs (females p = 0.213, males p = 0.054) or in hearing thresholds at individual frequencies between the patients and the reference values (Table 4). Three patients (7%), of whom two had hearing aids in both ears, had moderate hearing loss with their better ear PTA varying from 41 up to 48 dB HL. The hearing loss of one of them had been discovered already at an early age and the patient had received a hearing aids at the age of 40 years. No significant differences between air and bone conduction thresholds were detected, suggesting that there was no conductive hearing loss among the patients (Table 5). This corroborated the validity of the air-conduction thresholds obtained via automated audiometry.

**Table 4:** Statistical comparison of hearing thresholds at individual frequencies between AGel amyloidosis patients and the data derived from ISO7029 standard

Frequency (Hz)	Male	Female
	p**	p**
250	0.071	0.194
500	0.018	0.102
1000	0.147	0.147
2000	0.226	0.514
4000	0.049	0.314
8000	0.049	0.383

The statistical analysis was conducted using the Mann–Whitney U test

^**^p-values less than 0.0083 are considered statistically significant after the Bonferroni correction

**Table 5:** Mean pure-tone audiometry air-bone gaps in the 46 patients

Frequency (Hz)	Mean ABG (SD), right	Mean ABG (SD), left
250	0.9 (5.2)	0.2 (1.5)
500	2.1 (7.8)	3.3 (8.5)
1000	4.3 (9.3)	2.9 (8.2)
2000	0.2 (10.5)	0.5 (8.2)
4000	− 6.4 (11.0)	− 7.0 (9.9)
8000	0.8 (5.2)	0.7 (4.4)

ABG = air–bone gap, SD = standard deviation

### Speech recognition in noise

In some diseases involving the vestibulocochlear nerve, speech recognition is impaired, even though the pure tone audiogram may be normal [36]. To reveal such a possible retrocochlear hearing loss, the Finnish Matrix Sentence Test (FMST [32]) was performed for the AGel amyloidosis patients.

**Fig. 2:**
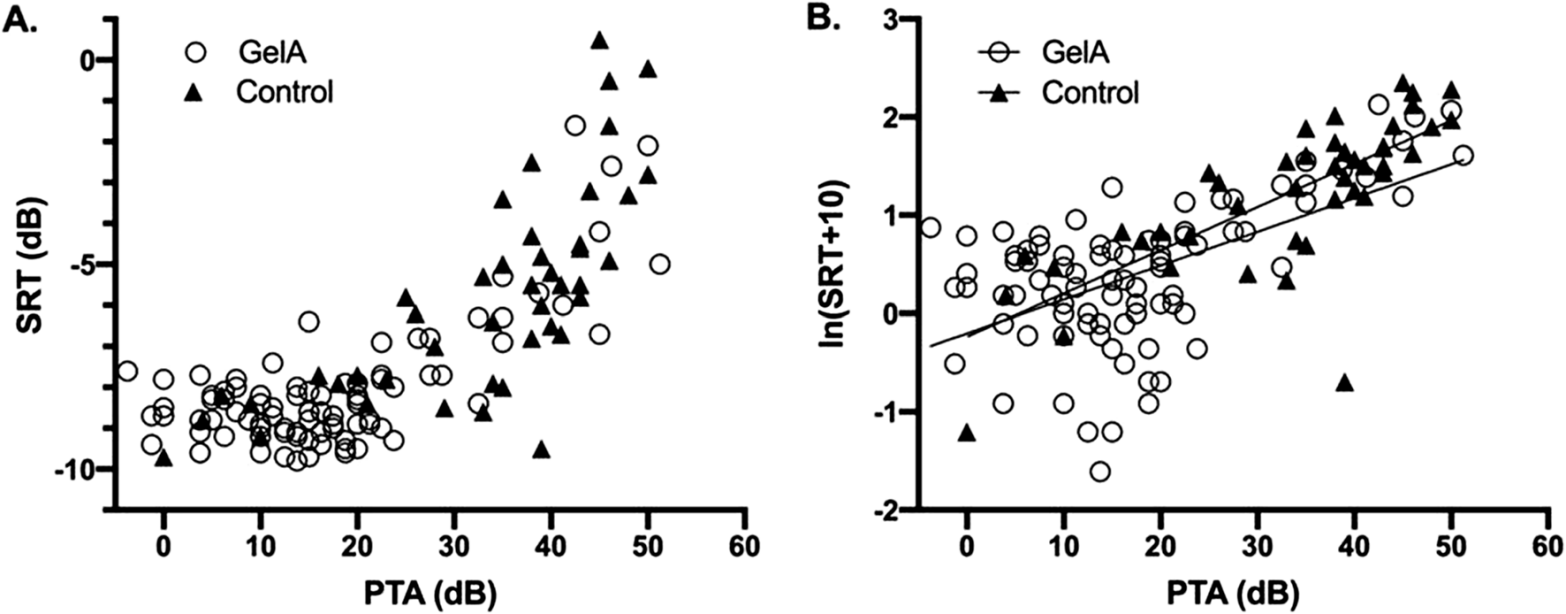
Speech reception threshold in noise (SRT) versus pure tone average (PTA) in AGel amyloidosis patients and PTA-matched controls. **A** SRT vs PTA (dB). **B** Ln(SRT + 10) vs PTA (dB) with linear regression models. * There was no statistically significant difference in between the slopes (F(1, 133) = 1,321, p = 0.253) and the intercepts (F(1, 134) = 3.445, p = 0.066) of the regression models for the two groups

The average speech recognition threshold in noise (SRT) was -8.2 (SD 1.5) dB signal-to-noise ratio (SNR) for the right and -8.0 (SD 1.7) dB SNR for the left ear. The relationship between SRT and PTA in AGel amyloidosis patients was similar to that of the control group with a comparable range in PTA (Fig. 2). After a logarithmic transformation, there was no statistically significant difference between the slopes (F(1,133) = 1,321, p = 0,253) or the intercepts (F(1, 134) = 3.445, p = 0.066) of the linear regression models for these two groups, suggesting that there is no evidence of abnormal functioning of the vestibulocochlear nerve in AGel patients.

### Hearing profiles in AGel amyloidosis patients reporting hearing problems

In order to compare hearing profiles of the patients reporting hearing impairment and those perceiving their hearing normal, a multivariate analysis of variance (MANOVA) between these groups and their results for the SSQ subscales, PTA and SRTs (Table 2) was performed. Even though the analysis resulted in significant difference between the groups (F(7, 35) = 5.294, p < 0.0005; Wilk's Λ = 0.486, partial η^2^ = 0.514), the effect of reported hearing loss was significant only in the subjective Speech and Qualities subscales of the SSQ. However, none of the objective measures in the entire study population indicate objective hearing loss regardless of whether the patient experienced a subjective hearing impairment or not.

## Discussion

In this study, the hearing problems associated with AGel amyloidosis were evaluated by means of self-report, the SSQ questionnaire, automated audiometry and a speech-in-noise test. Although the AGel amyloidosis patients often report subjective hearing impairment, in a more detailed self-assessment as well as in audiometric tests, hearing seemed to be in the normal range adjusted for the age of the patients.

In general, otological manifestations of amyloid diseases are extremely rare [24]. Amyloidoses manifesting in the head and neck region are typically local with good prognosis. Larynx is the most commonly affected site in local amyloidosis, while macroglossia is seen most frequently in a systemic disease [37]. Rarely, amyloid accumulation (due to local or systemic disease) is seen in the external ear as lesions in the auricle or obstructing masses of the external auditory canal [38–41]. Additionally, one case report of middle ear involvement has been published [42]. Recently, in cases of ATTRwt and ATTRv amyloidoses, hearing loss was found to be more prevalent compared to the general population [25, 26]. The authors speculated amyloid infiltration of the inner ear and in case of ATTRv, also of the middle ear to be the etiological cause. In AGel amyloidosis, however, the self-reported hearing impairment could be due to vestibulocochlear nerve dysfunction, since cranial neuropathy, unlike in other amyloid polyneuropathies [12], is a prominent manifestation of the disease. Reflecting this, substantial amyloid accumulation has been demonstrated in small facial nerve branches [43] which is, for unknown reasons, particularly affected in AGel amyloidosis. In an autopsy of an 82-year-old patient, amyloid was found in facial and trigeminal nerve roots, but not in other cranial nerve roots or intralingual nerves [44]. It is not known why cranial nerves VII and V are particularly affected in AGel amyloidosis. The clinical cranial nerve involvement is, however, widespread in AGel amyloidosis [1, 12, 14, 30].

The prevalence of self-reported hearing impairment in AGel amyloidosis varies from 27% up to 52% [12, 14, 20, 28], which is in line with the 39% noted in this study. The SSQ is a widely used self-assessment measuring the listener’s hearing abilities in situations encountered in everyday life [31]. Importantly, even though young, normal-hearing listeners do not rate their hearing ideal, the results are clearly worse among the hearing impaired [31, 34]. In the largest SSQ study using young normal-hearing listeners, the mean response ranged from ca. 7 to 8.5 (out of 10) in each of the sub-scales [34]. In a study comparing SSQ results between younger (mean age 19 yrs) and older (mean age 79 yrs) normal-hearing listeners, the average results were somewhat better (8.8 vs 7.7) among younger listeners [45]. In comparison, listeners with moderate hearing loss scored on average 5.5 [31]. In this study, nine patients evaluated their hearing abilities under 7 in one or several SSQ subscales, whereas most of them had relatively good results in the objective measurements (Table 3). Generally, the SSQ responses of AGel amyloidosis patients (ranging on average from 8.2 to 8.6) are at the same level as with normal-hearing young people despite the large proportion of self-reported hearing impairment.

The audiometric results of this study contradict the previous hypothesis of a disease-related hearing impairment detected in pure tone audiometry [27, 29, 30] – the most widely used hearing test. The mean PTA, representing hearing sensitivity at the key frequencies for speech, was in the normal range (17.1 dB HL for both ears) with no difference to the reference values. A PTA of 20 dB HL or less is defined as normal hearing by the World Health Organization. The median hearing threshold distribution in AGel amyloidosis patients resembles the typical shape of high frequency sloping loss seen in sensory presbyacusis [46], as well as in age and gender matched controls [35] (Fig. 1).

Finally, what comes to SRT, there was no difference between AGel amyloidosis patients and the control group with a comparable range in PTA (Fig. 2). As also previously reported in a sample of 177 patients with PTA (0.5, 1, 2, 3 kHz) ranging from − 5 to 90 dB HL, SRT increased with increasing PTA (Fig. 2A) [33]. The FMST [32] measures hearing abilities under background noise and is suitable for this study, as a large proportion of patients self-reported their hearing abilities in background noise compromised. As the FMST has been quite recently validated in Finnish and taken into clinical practice, there is only limited amount of normative data available. The difference between the mean SRT for the AGel amyloidosis patients (− 8.2 and − 8.0 dB SNR for the right and left ear, respectively) and the corresponding expected value of -9.7 ± 0.7 dB SNR for young normally hearing adults [32] appears to be relatively minor, especially given the higher age of the patients in this study. More importantly, when accounting for the average effect of PTA on SRT (Fig. 2), the hearing abilities of the patients in background noise did not deviate from the control group.

## Limitations

Firstly, this study describes hearing abilities in AGel amyloidosis patients aged 47 to 66 years and since the disease is age-related it is possible that audiologic manifestations appear later, at a more advanced stage of the disease. However, the prevalence of presbycusis increases strongly with age and this would be a major confounding factor with an older study population. All of the patients were clearly symptomatic due to the AGel amyloidosis and cranial neuropathy was observed in almost all of the patients (Table 1). A large proportion of the patients had also received surgical treatment, indicating an already advanced disease. Thus, in our opinion, the current study population is justified. Secondly, the number of patients (46) is limited but can still be considered as a representative sample, considering that AGel amyloidosis is a rare condition.

## Conclusions

While many AGel amyloidosis patients report subjective hearing problems, the SSQ, audiometry and speech-in-noise test showed no substantive indication of AGel amyloidosis-related hearing loss in an already advanced stage of the disease. With this knowledge, the clarification of a very multifaceted clinical picture of this rare disease takes one step forward—important for the treating physicians without undermining the importance of the matter for the patients.

## Methods

### Subjects

AGel amyloidosis patients aged between 50 and 66 years in the FIN-GAR registry were identified (n = 89) and invited by mail or informing of the current study at the annual patient organization meeting (Finnish Amyloidosis Association). Informed consent was obtained from each patient before the investigations. The local ethics committee and the institutional research review board of the Helsinki University Hospital approved the study.

The patients either completed the 48-item SSQ questionnaire in Finnish [47] at the appointment or took it home for completion.

All the patients were interviewed and clinically examined by the same physician (T. M.). General questions were asked regarding disease symptoms, including specific questions on hearing difficulties. Pneumatic otoscopy, tuning fork tests and thorough clinical examination of cranial nerves were performed.

### Audiometric evaluation

Pure-tone hearing thresholds were determined utilizing the Automated Method for Testing Auditory Sensitivity (AMTAS) run with a GSI AudioStar Pro clinical audiometer (Grason-Stadler, Eden Prairie, MN, USA). Air-conduction thresholds were obtained on every octave between 250 Hz and 8 kHz with a RadioEar DD450 circumaural headset, and when needed, bone-conduction thresholds between 500 Hz and 4 kHz with a RadioEar B81 bone conductor placed on the forehead under an elastic AMBAND headband. The self-administered AMTAS procedure uses masking noise always on the non-test ear and provides equivalent thresholds with manual audiometry in air-conduction testing [48]. PTA was calculated as the average of the hearing threshold at 0.5, 1, 2 and 4 kHz. The resulting hearing thresholds at each frequency and PTAs for the right and the left ear were compared with gender- and age-matched reference values published in the ISO 7029 (2017) standard [35]. Air–bone gap was calculated as the difference between air- and bone-conduction thresholds.

SRTs were measured for the patients separately for each ear with the FMST [32] using the Oldenburg Measurement Applications version 1.3 software (HörTech gGmbH, Oldenburg, Germany). The software was run on a laptop PC connected to an external RME Fireface 802 audio interface and circumaural Sennheiser HDA200 headphones. The speech material of the FMST comprises five-word sentences which are selected pseudorandomly from a 5 × 10 word matrix. The noise signal of the FMST is composed by superimposing the sentences multiple times such that the resulting noise is quasi-stationary, while it retains the long-term spectrum of the speech material. For more details on the international matrix sentence tests, see [49].

The clinical application of the FMST includes familiarization with the word matrix in writing, followed by a list of 20 sentences with the speech presented at 75 dB SPL and the noise at 65 dB SPL i.e., with a fixed + 10 dB SNR. This is followed by another list of 20 sentences with the noise at 65 dB SPL and the speech level is varied in an adaptive procedure which converges on a 50% level of correct responses. The familiarization accounts for short-term learning of the speech material [32], and any subsequent lists of 20 sentences with varying speech level measure the SRT, which is expressed in dB SNR. In this study, sound was presented to both ears in the familiarization phase, and the order of the right and left ear SRT measurements were counterbalanced across subjects. The obtained SRTs were then compared with a control group of non-AGel amyloidosis patients with a comparable range in PTA thresholds.

### Statistical analyses

The analyses were performed with IBM SPSS V25 software (IBM, Armonk, NY, USA). Based on the ISO7029 standard [35], reference values were generated for every fifth percentile resulting in 21 age- and gender matched audiograms, which were then compared with the audiograms obtained for the AGel amyloidosis patients with the Mann–Whitney U-test. The Bonferroni method was used to adjust for multiple comparisons.

Since the relation between PTA and SRT is nonlinear, a logarithmic transformation was first applied on the SRT data. Then a linear regression model was applied between PTA and SRT separately for the AGel amyloidosis patients and for the control group with a comparable range in PTA, and the equality of the slopes and the intercepts between the two linear regression models were tested using the F-test.

A multivariate analysis of variance (MANOVA) was applied on the SSQ questionnaire and audiometric data with the SSQ items, PTAs and SRTs as dependent variables and self-reported hearing problem as an independent variable. A univariate analysis of variance (ANOVA) with the Bonferroni correction for multiple tests was then applied separately to each dependent variable.

## Data Availability

The data used in this study is available from the corresponding author on reasonable request.

## Abbreviations

AGel: Gelsolin amyloid
AMTAS: The Automated Method for Testing Auditory Sensitivity
ANOVA: Analysis of variance
ATTRv: Hereditary transthyretin amyloidosis
ATTRwt: Wild-type transthyretin amyloidosis
FIN-GAR: The Finnish Gelsolin Amyloidosis Patient Registry
FMST: Finnish matrix sentence test
MANOVA: Multivariate analysis of variance
PTA: Pure tone average
SNR: Signal-to-noise ratio
SRT: Speech reception threshold in noise
SSQ: The Speech, Spatial and Qualities of Hearing Scale
